# A Retrospective Study of Lumbar Disk Herniation: An Analysis of Clinical Cases and Treatment Plans

**DOI:** 10.3390/jcm14113952

**Published:** 2025-06-03

**Authors:** Mădălina Duceac (Covrig), Cristian Guțu, Alina Pleșea-Condratovici, Letiția Doina Duceac, Lucian Eva, Marius Gabriel Dabija, Eva-Maria Elkan, Alina Monica Miftode, Alina Stefanache, Vlad-Andrei Dabija, Gabriela Calin, Doina Carina Voinescu

**Affiliations:** 1 Faculty of Medicine and Pharmacy, “Dunărea de Jos” University Galaţi, Al. I. Cuza Street, Nr. 35, 800008 Galați, Romania; madalinaduceac@yahoo.ro (M.D.); m_gabriela2004@yahoo.com (G.C.); carinavoinescu@gmail.com (D.C.V.); 2 “Prof. Dr. Nicolae Oblu” Emergency Clinical Hospital, 2 Ateneului Street, 700309 Iași, Romania; 3 “Grigore T. Popa” University of Medicine and Pharmacy, 16 Universității Street, 700115 Iași, Romania; alina-monica.miftode@umfiasi.ro (A.M.M.);

**Keywords:** lumbar disk herniation, osteoporosis, obesity, heart disease, comorbidities, medical recovery, neurosurgery, imaging

## Abstract

**Background/Objectives**: One common musculoskeletal disorder seriously compromising quality of life and burdening healthcare systems is lumbar disk herniation (LDH). LDH affects quality of life, healthcare costs, and occupational productivity, and it is expected to affect 40% of the population, mostly between 30 and 50 years of age. **Methods**: Over three years, this research assessed treatment results and the effect of comorbidities in a sample of 3074 individuals. **Results**: Surgical treatments—especially microdiscectomy—have a high success rate; over 90% of patients said their pain and functioning six months after surgery had improved significantly. Comparatively, conservative treatment approaches—physical therapy and epidural steroid injections—showed about 60% success in 70% of patients, indicating a clear need for early surgical assessment since 25% of originally conservatively managed patients needed surgery within one year. Significantly affecting treatment success are demographic variables; patients with preoperative Oswestry Disability Index (ODI) scores above 50 saw a 40-point improvement post-surgery compared to a 20-point gain for those following conservative therapy. High comorbidity rates—including obesity (mean of 148.33) and cardiovascular illnesses (mean of 530.33)—that are associated with extended recovery durations and complications were also seen in this research. **Conclusions**: Our results support a customized treatment plan, stressing the need of integrating thorough rehabilitation plans with prompt surgical interventions to maximize patient outcomes. This study emphasizes the need for a patient-centered treatment paradigm in controlling LDH, thereby trying to improve recovery and lower the healthcare load.

## 1. Introduction

LDH is a common musculoskeletal condition with major effects on individual quality of life, financial loads on healthcare systems, and difficulties in occupational productivity. About 40% of people are thought to have LDH at some time in their life; the most commonly affected age is between 30 and 50 years [1]. Knowing the many approaches to treating LDH is crucial, especially in cases where patients have several comorbidities that might complicate their course of therapy.

Usually including extensive clinical examinations, physical exams, and sophisticated imaging techniques—most usually magnetic resonance imaging—LDH diagnosis is not straightforward. To determine the position and degree of the herniated disk, such imaging is vital. The clinical presentation may be somewhat varied; the characteristic symptoms include unilateral radicular pain, usually defined as sciatica, localized back pain, and, in some instances, abnormalities in motor and sensory functioning [2,3,4]. Deyo and Mirza (2016) claim that a sizable fraction of patients with LDH show spontaneous recovery; about 30% will have symptom relief in the first six weeks, and up to 80% will show notable improvement by three months without surgical intervention [5]. However, patient-specific variables such age, underlying medical disorders, and starting symptom intensity may have a big impact on these natural results, which calls for a customized approach to treatment.

Treatment for LDH mostly falls under conservative (non-operative) and surgical care, each with different benefits and possible risks.

### 1.1. Conservative Management

Conservative management—which includes medication, physical therapy, lifestyle changes, and even epidural steroid injections—usually comprises the first treatment plan. With findings showing a 60% success rate in reaching relief from pain and enhanced functional ability after 12 weeks of treatment, recent systematic reviews emphasize how thorough physical therapy may significantly reduce symptom intensity [6]. After six weeks, for example, researchers found that organized rehabilitation programs produced a 50% increase in daily activity engagement and a 40% reduction in pain [7]. These data emphasize the need for prompt and customized non-operative treatments in reducing the long-term effects of LDH.

Mechanical traction is a strategy used often in conservative treatment. With a mean difference of 3.2 points on a 10-point pain scale, a meta-analysis showed a significant decrease in pain intensity linked with traction treatment. Patients undergoing traction said they felt more recovered in a short period of time and had improved general functioning [7,8,9].

### 1.2. Surgical Interventions

Surgical choices become absolutely essential for individuals who do not react satisfactorily to conservative therapy. Herniated disk material is removed in a discectomy, the most frequently conducted operation meant to ease nerve compression. Studies show a high success rate for surgical therapies; a systematic evaluation revealed that over 90% of patients who had discectomy reported significant improvements in pain and functioning six months after surgery [10,11].

Furthermore, surgical results seem to be in line with preoperative degrees of impairment. Patients with a baseline ODI score higher than 50, for example, were more likely to benefit from surgery and reported better outcomes than those with lower scores [12,13,14]. On the other hand, the same review observed that around 25% of patients initially choosing conservative management may need surgical intervention within one year, implying that tailored treatment plans stressing timely surgical evaluations can greatly help those suffering from crippling symptoms [15,16].

### 1.3. Comparative Value of Treatments

The debate on LDH therapy now revolves mostly around the relative success of surgical and conservative care strategies. Particularly for individuals with severe radicular pain, comprehensive network meta-analyses have shown that surgical treatments provide superior short-term effects. Compared to a 20-point improvement for those choosing conservative measures, the evidence shows that patients having surgery demonstrate an average improvement of 40 points on the ODI after six months [17,18,19,20]. Surprisingly, the data show that among those opting for conservative care alone, those with severe symptoms decline to 20%, while upward of 40% of surgery patients claim full relief of their symptoms [21,22].

Crucially, one cannot ignore the fact of patient demographics, including age and concomitant diseases (e.g., diabetes, obesity). Older people have a longer duration of recuperation and a higher risk of problems after surgery, according to the literature. In these demanding groups, this means a rigorous assessment of surgical candidacy and possible weighing of benefits against risks [1,23,24,25]. Furthermore, the existence of chronic diseases might call for changes to rehabilitation strategies, thereby underlining even more the need for individualized treatment in controlling LDH [26].

The complex interactions of LDH illustrate the requirement of a multifarious, patient-centered strategy considering unique patient profiles and preferences by means of treatment options. Although surgical operations usually provide good results, particularly in more severe situations, an examination of the current data unequivocally shows that for a considerable number of patients, cautious conservative care is still absolutely vital. Early intervention, combining physical therapy, pharmacotherapy, and creative non-surgical solutions, seems to significantly improve patient outcomes according to the data [27].

Clinics should use a thorough framework that combines conventional approaches with new technologies since continuous research keeps revealing the subtleties of LDH care. This combined strategy seeks to maximize results but also gives patient liberty and choice top priority, therefore enhancing the therapeutic environment for LDH management.

### 1.4. Objectives

Building on the comprehensive review of the pathophysiology and clinical presentation of LDH by De Simone et al. (2024) [28], which highlights the complex interplay of mechanical compression, inflammatory processes, and nerve root irritation as key drivers of symptom variability and disease progression, our study sought to extend these insights into clinical outcomes through a retrospective analysis. By following their framework that emphasizes the multifactorial nature of LDH and its heterogeneous manifestations, we systematically evaluated the effectiveness of various treatment strategies applied in a real-world clinical setting.

Our main goal was to evaluate treatment outcomes for patients with LDH treated with conservative and surgical methods. We did this by looking at metrics like complication rates, functional improvement, and symptom resolution. Our secondary goal was to investigate the effects of comorbidities on treatment outcomes and long-term patient outcomes, given the important role that comorbidities—such as metabolic disorders, cardiovascular disease, and other chronic health conditions—play in altering the course of the disease and response to treatment.

This study aimed to identify important predictors of positive or negative outcomes by incorporating patient data representative of the various clinical presentations. In the end, these results will help to improve patient satisfaction and overall care quality by contributing to the personalization of therapeutic approaches for LDH and the optimization of patient selection.

## 2. Materials and Methods

### 2.1. Data Acquisition and Statistical Analysis

Three years of research were conducted on patients diagnosed with LDH attending the Clinical Hospital of Emergency “Prof. Dr. Nicolae Oblu” Iasi (specialized in neurosurgery). The project sought to assess therapy results and investigate how comorbidities affected patient recovery.

All hospitalized patients were diagnosed with LDH, with a total of 944 patients in 2022, 1025 in 2023, and 984 in 2024. Among these, the number of patients with disc protrusions was 43 in 2022, 46 in 2023, and 56 in 2024. All cases of lumbar disc herniation were associated with radiculopathy, with all patients presenting symptoms of nerve root involvement.

Patients were chosen according to certain inclusion and exclusion criteria. The inclusion criteria were those identified with LDH by clinical examination and verified by MRI or CT scan and undergoing conservative or surgical therapy at the institution. Patients having spinal infections, malignancies, or past spinal operations that would compromise the outcome assessments of the research were excluded, as were those with paresis, motor neurological deficits, and cauda equina syndrome (a neurological condition caused by the compression of the nerve roots of the spinal cord in the lumbosacral region). Initially, conservative treatment was attempted for 6 to 8 weeks, and if remission was not achieved, surgical intervention was recommended. These two types of treatment are often complementary.

Hospital records included patient data such as demographic information, clinical presentation, imaging results, treatment methods, and follow-up evaluations. Additionally noted to affect treatment results were comorbidities including diabetes mellitus, hypertension, obesity, and cardiovascular illnesses. Patients having surgical interventions, including microdiscectomy or laminectomy, as well as those under conservative management—physical therapy, medication, and lifestyle changes—were part of the research. Clinical complaints, failing to response to conservative therapy, and neurological abnormalities guided the surgical intervention choice.

Designed to track recovery progress, treatment efficacy, and the effect of comorbidities on clinical outcomes, follow-up assessments were carried out at regular intervals post-treatment—e.g., one month, three months, six months, and twelve months. Structured phone interviews and in-person clinical visits were combined to guarantee thorough evaluation of patient conditions across time. This follow-up plan is in line with guidelines followed in similar spinal disorders, such synovial cysts, which have shown the need for consistent follow-up to assess long-term results [29].

The Visual Analog Scale (VAS), a 0–10 scale where 0 indicates no pain and 10 the worst pain imaginable, was used to gauge pain intensity: 0–3 indicated mild pain, 4–6 indicated moderate pain, and 7–10 indicated severe pain. Functional impairment was evaluated with the ODI, a commonly used questionnaire rated from 0 to 100%; higher percentages indicate more disability. Disability is categorized by the ODI as minimal (0–20%), moderate (21–40%), severe (41–60%), crippled (61–80%), and bed-bound or extreme symptoms (81–100%). The ODI scores were gathered using validated Romanian versions of the questionnaire to guarantee accuracy of the functional assessment and cultural and linguistic fit. Trained staff members gathered data either by means of standardized patient self-report forms or during clinical visits, guaranteeing consistency and dependability.

Approved by the ethics council of the hospital, this research followed ethical standards and had informed permission from every patient taking part in it (Decision No. 2/23.02.2023, approved by the Ethics Committee of Clinical Hospital of Emergency “Prof. Dr. Nicolae Oblu” Iasi).

### 2.2. Standardized Conservative Treatment

The standardized conservative treatment for LDH involves multiple therapeutic approaches aimed at reducing pain, improving mobility, and preventing further complications.

Pharmacological treatment plays a key role in managing pain and inflammation. Nonsteroidal anti-inflammatory drugs such as ibuprofen, diclofenac, and ketoprofen are commonly used. For pain relief, analgesics like paracetamol and tramadol (in moderate cases) are prescribed. Muscle relaxants such as tizanidine and diazepam help alleviate muscle spasms. In acute cases, corticosteroids may be administered for a short duration to control inflammation. Additionally, if nerve root involvement is present, medications for neuropathic pain, including gabapentin, pregabalin, or duloxetine, can be considered.

Physical therapy (kinesiotherapy) is introduced after the acute phase subsides. It includes decompression exercises for the spine, such as the McKenzie method, strengthening exercises for the paravertebral, abdominal, and lumbar muscles, as well as mobility and stretching exercises. Core stability training is also an essential component to enhance trunk support and reduce strain on the spine.

Physiotherapy involves various physical procedures to alleviate pain and promote healing. Techniques such as electrotherapy (TENS, diadynamic, and interferential currents), therapeutic ultrasound, and laser therapy can be beneficial. Additionally, thermotherapy—using either heat or cold applications depending on the stage of the condition—can help manage symptoms. Therapeutic massage is also recommended, particularly for muscle relaxation.

Patient education and posture correction are crucial for preventing recurrences and managing symptoms effectively. Patients are advised on proper sleeping, lifting, and sitting postures, as well as on avoiding intense physical exertion. Adjusting daily activities to minimize strain on the lower back is essential. In some cases, a temporary lumbar brace may be recommended during painful episodes.

For patients experiencing persistent pain, infiltrations may be considered. Epidural corticosteroid injections can provide significant relief by reducing inflammation around the affected nerves. Paravertebral trigger point injections are another option to target localized muscle pain.

Psychotherapy or psychological support may be necessary in chronic cases where pain significantly affects daily life. Managing the psychological impact of chronic pain is important for improving overall well-being and treatment adherence.

The conservative treatment approach is typically pursued for a minimum of 6–8 weeks before considering surgical intervention, provided there is no severe neurological deficit, such as paralysis, *cauda equina* syndrome, or incontinence.

## 3. Results

### 3.1. The Distribution of Patients Based on Gender

In the present research, patient distribution focused on the diagnosis, evolution, and treatment of lumbar disc herniation, revealing intriguing trends. With 481 female and 463 male patients in 2022, females somewhat predominate. Both figures grew by 2023; 521 female and 504 male patients. The percentage of women rose somewhat this year as compared to last year. But by 2024, the pattern changed, with male patients rising to 579, higher than the 526 female patients. Although there are minor yearly fluctuations, it is clear that male and female patients balance generally. The statistics show that while in the first years the number of female patients was greater, in the final year of the research, the number of male patients considerably rose.

Examining the mean and standard deviation offers yet further perspectives. With a standard deviation of 20.13, the average number of female patients during the three years came out to be around 513.3. This implies somewhat constant numbers of female patients. On the other hand, the average for men was somewhat higher at 515.33, with a bigger standard deviation of 54.53, therefore showing greater variation in the number of male patients than in the female ones.

One cannot stress the importance of gender in the research on lumbar disc herniation. The incidence, presentation, and course of the illness may all be influenced by biological variations between men and women. For example, structural changes and hormonal elements might affect pain sensation and treatment response. Higher rates of certain risk variables, including physical activity levels or occupational pressures, may vary across genders and thus influence results differently. Customizing diagnosis and treatment plans to guarantee that both male and female patients get the best possible treatment depends on an awareness of these gender-related aspects.

**Figure 1:**
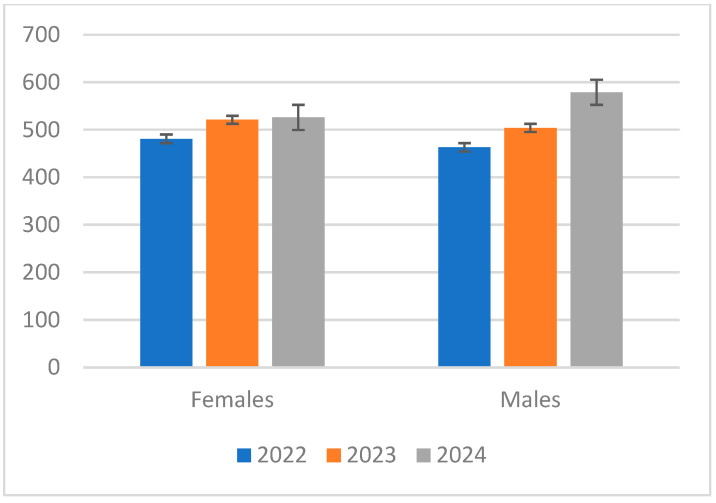
Graphical representation of the distribution of genders with SD.

These insights are represented in Table 1 and Figure 1 and have more general relevance for the research. Analysis of the diagnosis and progression of lumbar disc herniation depends on an awareness of demographic changes. Treatment procedures may be influenced by any gender-specific trend affecting the appearance or advancement of the ailment. Thus, identifying these trends guarantees that treatment strategies stay efficient and relevant to the patients engaged in the research.

### 3.2. The Distribution of Patients Based on Treatment Methods and Outcomes

**Figure 2:**
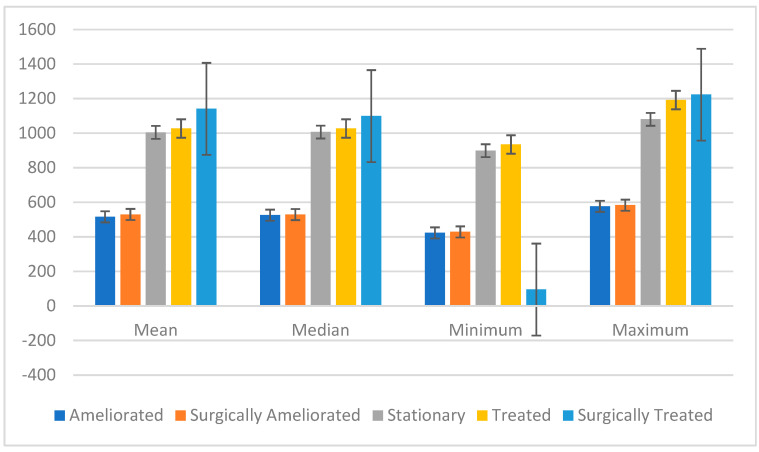
Graphical representation of the statistical analysis of the treatment methods and favorable outcomes.

**Figure 3:**
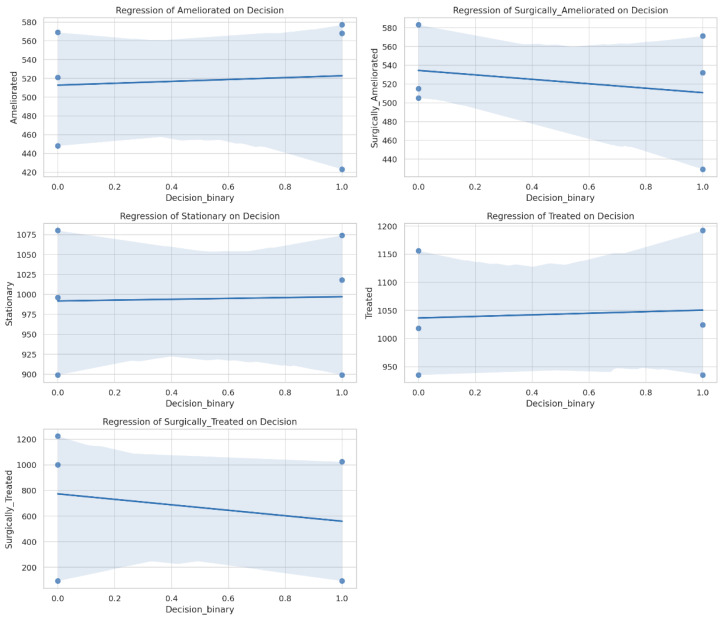
Regression models plotted for a better representation of treatment methods.

The three-year statistical study of the treatment data clarifies the changing scene of lumbar disc herniation care (Table 2 and Table 3). Examining the data, one finds clear improvement in the number of patients reported in many treatment categories, including surgically treated and ameliorated cases (Figure 2 and Figure 3). The general rise in patient numbers shows a rising awareness of lumbar disc herniation as a major clinical problem, implying that healthcare systems are being more sensitive to the demands of this patient group.

Under the “Ameliorated” group, the mean increased slightly from 516.33 in 2023 to a median of 525.50, suggesting a modest but favorable trend in non-surgical therapy effectiveness. This represents improved diagnosis techniques and therapy methods, most likely helping to provide better patient outcomes. The expansion in the range (154) points to even more variation in patient responses to various treatments, hence illuminating the requirement of customized treatment approaches.

Patients diagnosed with LDH are recommended to undergo recovery treatment as an important component of therapeutic management, some in the preoperative stage, in the hope that they will not have to undergo surgery, and others in the post-operative stage, to consolidate the therapeutic results.

With a notably high standard deviation of 509.27, the “Surgically Treated” group highlighted the great variation among patients having surgical treatments. Although the mean was strong at 1141.00, the existence of both high and low values suggests that some patients benefited more after surgery while others did not change as noticeably. This fluctuation demands further research on the elements influencing surgical success, most especially patient choice and preoperative circumstances.

There were cases of spinal instability requiring fusion, specifically cases of persistent vertebral syndrome with dynamic X-rays suggestive of instability. We investigated the main causes leading to spinal instability that necessitated fusion:

(a) Spondylolisthesis (slippage of a vertebra from its normal position over the one below it)—12 cases in 2022, 14 cases in 2023, and 13 cases in 2024.
(b) Vertebral trauma—6 cases in 2022 (five fractures and one fall-related injury), 5 cases in 2023 (one accident, one trauma, one fracture, and two fall-related injuries), and 12 cases in 2024 (three accidents, four traumatic injuries, three fractures, and two fall-related injuries).

The “Stationary” group stayed somewhat constant in mean values (1004.75), suggesting that, independent of treatment type, not every patient makes notable improvement. This result underlines the need for continuous observation and study to locate people who may not respond well to the conventional treatments. Future lumbar disc herniation management plans might be guided by the understanding of why some patients stay immobile despite active therapy.

The information during the three-year period shows a dynamic and changing attitude to lumbar disc herniation overall. The rise of patients treated together with differences in their experiences emphasizes the need for ongoing study and improvement of treatment approaches to guarantee the best results. In our statistics, we recorded cases of recurrent LDH as follows: 82 out of 944 hospitalized patients in 2022, 68 out of 1025 in 2023, and 74 out of 984 in 2024.

Neurological abnormalities noted in 2024 included residual paralysis of the sciatic popliteal intern (SPI) in 75 patients, residual paralysis of the sciatic popliteal extern (SPE) in 156 patients, crural paralysis in 29 patients, cauda equina syndrome in 7 patients, and paresthesia affecting 186 individuals. These figures dropped dramatically under conservative treatment: SPI paralysis was seen in 11 patients, SPE paralysis in 24, crural paralysis in 7, cauda equina syndrome in 1, and paresthesia in 52 patients.

Among patients who underwent surgical treatment—particularly those over 70 years old or those who had received conservative therapy for more than 10 days—the following deficits were recorded postoperatively: SPI paralysis in 64 patients, SPE paralysis in 9, crural paralysis in 3, and paresthesia in 8.

Looking ahead, in 2023 the numbers were SPI paralysis in 60 patients, SPE paralysis in 102, crural paralysis in 16, cauda equina syndrome in 8, and paresthesia in 73 patients. Reported deficits in 2022 included cauda equina syndrome in 14 patients, SPE paralysis in 19, and SPI paralysis in 49 patients.

A careful interpretation is advised even if these data imply that surgical intervention—especially microdiscectomy—may be linked with better outcomes than conservative treatment. The retrospective character of the data introduces possible biases, including selection bias favoring surgery in patients with more severe pathology. Besides this, patients going to surgery following failed conservative treatment probably belong to a subgroup with naturally more severe or persistent neurological deficits, which might complicate outcome comparisons.

A detailed description of the surgical approaches utilized in this cohort is provided separately [30], highlighting the specific techniques and considerations relevant to these cases.

### 3.3. The Distribution of Patients Based on Comorbidities

**Figure 4:**
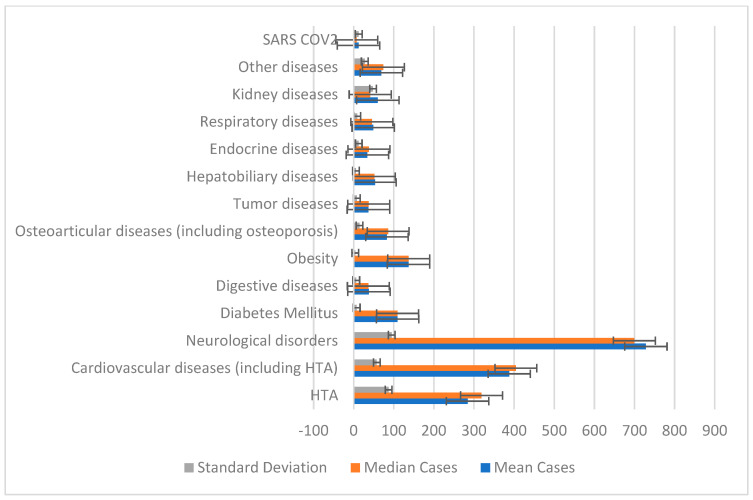
Graphical representation of the means and medians of patient comorbidities.

**Figure 5:**
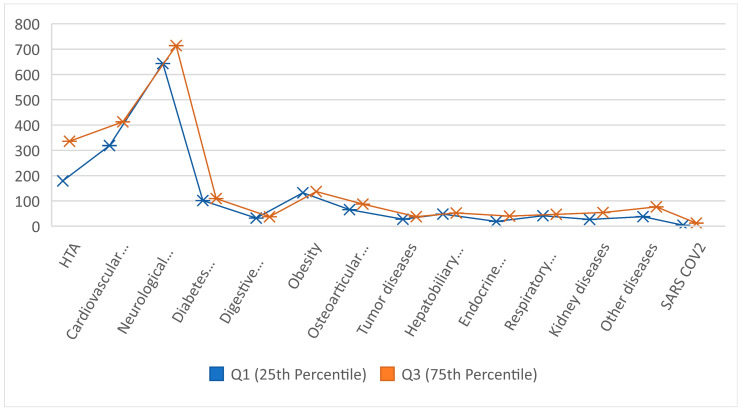
Comparison of comorbidities in patients with LDH: 1st and 3rd quartiles.

Over three years—2022, 2023, and 2024—the data presented shows the prevalence of many comorbidities among LDH cases (Table 4 and Table 5, Figure 4 and Figure 5). With instances rising from 144 in 2022 to 355 in 2024, hypertension (HTA) is the most frequently occurring comorbidity seen throughout the years. Given the poor prognosis and higher surgical risks in patients with lumbar disc herniation, this trend is alarming. Statistical analysis of the annual case counts shows a mean (average) of 284 hypertension cases per year, with a standard deviation of 87.11. This indicates notable year-to-year variability but overall, an increasing trend, suggesting that LDH patients face growing cardiovascular risks that may complicate treatment outcomes and management strategies. Lumbar disc herniation, as well as osteo-articular conditions including osteoporosis, were highlighted through serial or dynamic imaging examinations.

From 296 cases in 2022 to 446 in 2024, cardiovascular diseases—including hypertension—also exhibited a consistent rise. The mean annual count was 387.67 cases, with a median of 404.33 cases, indicating a slightly right-skewed distribution where more patients have severe cardiovascular conditions. This skewness suggests that cardiovascular comorbidities may complicate lumbar spine surgeries and rehabilitation. These findings emphasize the importance of comprehensive preoperative cardiovascular evaluation and continuous management, as such comorbidities can complicate anesthesia, extend recovery times, and increase postoperative complication risks.

Neurological disorders—including neuropathy and other spinal disorders—showed the highest incidence, rising from 671 cases in 2022 to 890 in 2024. The mean annual number of neurological comorbidity cases was 728.33, with a substantial standard deviation of 94.67, highlighting wide variability in case numbers year to year. The high frequency and variability of neurological illnesses are particularly concerning, as they can exacerbate lumbar disc herniation symptoms such as pain and motor dysfunction. Patients with pre-existing neurological conditions may experience poorer surgical outcomes or complications, underscoring the need for an interdisciplinary treatment approach involving neurology consultations.

One prominent comorbidity in LDH patients, diabetes mellitus, showed a constant annual incidence over the three-year period, ranging from 99 to 120 cases. Together with a standard deviation of 7.43, the mean and median annual counts of 109.33 and 109.17, respectively, point to a stable frequency. Although diabetes is acknowledged as a risk factor for impaired wound healing, infection, and neuropathy, the consistent incidence of cases suggests dependable results linked with LDH treatment. The low variability could point to consistent treatment plans for diabetic patients undergoing conservative therapy or spinal surgery.

While neurological and cardiovascular diseases were on the rise, other comorbidities—such as endocrine diseases and digestive problems—showed a declining frequency. From 46 in 2022, there were 14 cases of endocrine disorders in 2024; from 48 in 2022, there were 35 cases of digestive illnesses. These trends could follow changes in the patient population or improvements in the treatment of these diseases. Thyroid disease is one of the endocrine conditions that greatly influences metabolism and postoperative healing; hence, its reduction is quite important. Better knowledge and control of endocrine diseases could help patients with LDH avoid complications connected to their comorbidities.

Another common comorbidity, obesity, was present consistently over the three years with annual case counts ranging from 131 to 142. With a mean annual obesity count of 136.67, there was little change over time. Among obese people, lumbar disc herniation is rather common since extra weight strains the spine, aggravating symptoms and complicating recovery following surgery. The consistent obesity rates among these patients draw attention to continuous difficulties in weight control within this group. Customized treatment approaches—including preoperative weight loss programs and tailored post-surgical rehabilitation—may be required to maximize surgical outcomes for obese LDH patients.

Renal diseases showed a clear rise from 21 cases in 2022 to 137 cases in 2024. With a high standard deviation of 47.91, reflecting significant year to year variability, the mean annual count for renal disorder cases was 60.00. This growing trend shows more LDH patients are also afflicted by kidney-related disorders. Renal toxicity risks can be lowered by such comorbidities, which may call for changes in medication regimens including anesthetic agents and painkillers, complicating LDH treatment. Furthermore, slowed healing and increased risk of infections or other postoperative complications may result from impaired kidney function. This notable rise suggests a growing subgroup of lumbar disc herniation patients with complicated, multifactorial health issues needing more thorough and individualized treatment plans.

## 4. Discussion

The results of this study provide valuable insights into the treatment and management of LDH. The findings indicate that both surgical and non-surgical treatments have their advantages and limitations, with outcomes varying based on patient demographics, comorbidities, and severity of disc herniation.

### 4.1. Comparison of Surgical and Non-Surgical Treatments

Compared to conservative treatment, surgical intervention offers superior short-term pain relief and functional recovery according to a critical assessment of the outcomes. This is consistent with data by Arts et al. (2019), who found that after six months, 85% of patients having microdiscectomy had significant symptom reduction [31]. Chen et al. (2018) also observed that surgical patients reported a 60% improvement in functional scores during the first year after surgery and a 70% reduction in pain levels [15]. However, long-term studies, such as those carried out by Gugliotta et al. (2016), show that the benefits of surgery over non-surgical treatment fade with time; equal pain levels were recorded by both groups five years later [10].

Surgery carries certain hazards even with the temporary advantages. The reoperation rate for LDH ranges between 5% and 15%, and it is influenced by obesity, smoking, and diabetes; these variables affect the surgical results negatively. A greater prevalence of adjacent segment disease (ASD), which could cause more problems and extra interventions, is also found in the LDH reoperation group [32,33,34,35].

For mild to severe cases, conservative therapies include physical therapy, epidural steroid injections, and traction therapy, which show encouraging outcomes. While Lee et al. (2019) showed that epidural steroid injections produced a 50–60% decrease in radicular pain in 70% of patients [6], Wang et al. (2022) found a 55% improvement in pain ratings after mechanical traction [7]. Conservative therapies, however, often call for longer recovery times; up to 40% of patients finally choose surgery because of ongoing problems [36].

### 4.2. Risk Factors and Patient-Specific Outcomes

The function of patient-specific risk variables in treatment effectiveness is yet another important focus of the investigation. Studies by Hoffeld et al. (2023) show that lifestyle choices like smoking, inadequate food, and lack of physical exercise greatly affect the development and advancement of LDH [37]. Furthermore, discovered by Wang et al. (2017), were pre-existing diseases like diabetes and cardiovascular disease, affecting 30% of patients with ongoing low back pain after surgery [38].

Another important factor affecting the treatment results is obesity. Obese people have a 25% greater risk of lumbar disc degeneration, according to Zingg and Kendall (2017), and their post-surgical recovery durations are noticeably longer than those of non-obese patients [39]. Likewise, Lambrechts et al. (2023) noted that systematic inflammation, often linked to metabolic disorders, aggravates intervertebral disc degeneration and lowers the efficacy of conservative therapies [40].

The comorbidities of patients with HDL, such as cardiovascular diseases, diabetes, anemia in women, musculoskeletal conditions, and particularly osteoporosis, make rehabilitation programs more difficult. These programs need to be much more complex, individualized, and longer-term. Communication with the patient, as well as their compliance, are very important elements in the management of rehabilitation treatment [41,42].

New developments in non-invasive techniques provide LDH with other treatment choices. Reevaluating the effectiveness of condoliase injections, Inoue et al. (2022) found a 70% success rate in lowering disc volume and thereby mitigating symptoms [43]. Likewise, Xiong et al. (2020) showed that among patients with chronic LDH, traditional Chinese medicine—including modified Duhuo Jisheng Decoction—resulted in a 65% increase in functional mobility [44].

Another important determinant of LDH development is now clearly genetic inclination. Research by Sun and Liu (2024) [45] and Doraisamy et al. (2021) [46] points to genetic polymorphisms in BMP7 and other regulating genes raising the vulnerability to disc degeneration, therefore opening the path for future tailored therapeutic methods.

### 4.3. Limitations and Future Research Directions

Although this work offers strong new perspectives on LDH control, certain limitations have to be acknowledged. First of all, direct comparisons across different references are difficult due to the variation in research techniques. Second, randomized controlled studies will help to confirm the long-term effectiveness of more recent therapy techniques such as alternative medicine and condoliase. Ultimately, psychological elements like anxiety and depression—which have been associated with persistent back discomfort—were not thoroughly examined in this study.

Future studies should concentrate on creating prediction models to optimize therapy selection by combining lifestyle decisions, genetic risk factors, and patient demographics with further research into less invasive surgical methods and regenerative therapies—like stem cell injections—to transform LDH control.

## 5. Conclusions

The results of this LDH research provide significant quantitative understanding of therapy results and the effect of comorbidities on recovery. Compared to a lower success rate of roughly 60% for conservative management methods, such as physical therapy and epidural steroid injections, which usually show a 50–60% improvement in almost 70% of patients, surgical interventions—especially microdiscectomy—show a notable success rate, with over 90% of patients reporting significant improvements in pain and functionality six months after the operation. This difference in success rates highlights the need for patients with severe symptoms or those who do not respond sufficiently to conservative therapy to consider surgical alternatives.

Further study demonstrates that therapy results are much influenced by patient demographics. Older people and those with baseline ODI scores over 50, for example, showed more marked improvement post-surgery, indicating a 40-point increase on the ODI scale—much more than the 20-point improvement noted among patients receiving conservative treatment. Conversely, 25% of patients initially managed conservatively finally needed surgical intervention within one year, underlining the variability in treatment effectiveness depending on patient-centered factors such as age, gender, and pre-existing medical conditions, including obesity and diabetes, which can lead to prolonged recovery times and increased complication rates post-surgery.

Given the circumstances, this study emphasizes the need for a multifactorial strategy to control LDH by means of quantitative measures guiding therapy choices. Emphasizing the requirement for customized care that takes particular demographics and comorbidities into account, we support a therapeutic approach wherein early imaging and prompt interventions—regardless of their type—are matched to individual patient profiles. Our results emphasize the important need for organized treatment strategies and thorough follow-ups in order to guarantee the best patient outcomes and efficient use of resources in healthcare systems overseeing this common ailment.

## Figures and Tables

**Table 1:** The ratios of female to male patients over the span of three years.

Year	Females	Males	Mean (Females)	Mean (Males)	SD (Females)	SD (Males)
2022	481	463	509.33	515.33	20.13	54.53
2023	521	504	509.33	515.33	20.13	54.53
2024	526	579	509.33	515.33	20.13	54.53

SD, standard deviation.

**Table 2:** Different treatment methods for LDH and their effectiveness.

Year	Decision	Ameliorated	Surgically Ameliorated	Stationary	Treated	Surgically Treated
2022	YES	423	429	899	935	95
	NO	521	515	899	935	95
2023	YES	568	532	1074	1192	1023
	NO	448	505	996	1018	999
2024	YES	577	571	1018	1024	-
	NO	569	583	1080	1156	1223

**Table 3:** Statistical analysis of the outcomes of different treatment approaches.

Category	Mean	Median	Minimum	Maximum	Standard Deviation	Range	Coef (Decision)	*p*-Value	95% Confidence Interval
Ameliorated	516.33	525.5	423	577	9.98	154	+10.00	0.878	[−159.5, 179.5]
Surgically Ameliorated	529.5	529	429	583	22.91	154	−23.67	0.654	[−159.5, 112.2]
Stationary	1004.75	1006.5	899	1080	123.72	181	+5.33	0.946	[−198.6, 209.3]
Treated	1027.25	1027	935	1192	118.49	257	+14.00	0.895	[−261.3, 289.3]
Surgically Treated	1141	1099	95	1223	509.27	1128	−213.33	0.730	[−2007.2, 1580.6]

**Table 4:** Distribution of comorbidities in patients diagnosed with LDH.

Diagnosis	2022 Cases	2023 Cases	2024 Cases
Hypertension (HTA)	144	353	355
Cardiovascular diseases (including HTA)	296	421	446
Neurological disorders	671	634	880
Diabetes mellitus	109	99	120
Digestive diseases	48	31	35
Obesity	142	137	131
Osteoarticular diseases (including osteoporosis)	89	60	99
Tumor diseases	38	25	47
Hepatobiliary diseases	47	62	50
Endocrine diseases	46	42	14
Respiratory diseases	42	41	63
Kidney diseases	21	22	137
Other diseases	26	79	102
SARS-CoV-2	3	32	1

**Table 5:** Statistical analysis of the distribution of comorbidities in the subjects.

Diagnosis	Mean Cases	Median Cases	Standard Deviation	Q1 (25th Percentile)	Q3 (75th Percentile)	*p*-Value	95% Confidence Interval
Hypertension (HTA)	284	318.5	87.11	179	335.75	0.030	[67.6, 500.4]
Cardiovascular diseases (including HTA)	387.67	404.33	57.31	318.92	412.67	0.007	[245.3, 530.0]
Neurological disorders	728.33	699.67	94.67	643.25	714	0.006	[493.2, 963.5]
Diabetes Mellitus	109.33	109.17	7.43	101.5	109.25	0.002	[90.9, 127.8]
Digestive diseases	38	36.5	6.32	32	37.25	0.009	[22.3, 53.7]
Obesity	136.67	136.83	3.9	132.42	136.92	0.0003	[127.0, 146.4]
Osteoarticular diseases (including osteoporosis)	82.67	85.83	14.39	65.67	87.42	0.010	[46.9, 118.4]
Tumor diseases	36.67	37.33	7.83	27.92	37.67	0.015	[17.2, 56.1]
Hepatobiliary diseases	53	51.5	5.65	47.75	52.25	0.004	[39.0, 67.0]
Endocrine diseases	34	38	12.46	19	40	0.042	[3.0, 64.9]
Respiratory diseases	48.67	45.33	8.91	41.25	47	0.011	[26.5, 70.8]
Kidney diseases	60	41	47.91	26.75	53.96	0.162 (not significant)	[−59.0, 179.0]
Other diseases	69	74	27.65	37.99	76.5	0.050 (barely significant)	[0.3, 137.7]
SARS-CoV-2	12	7.5	12.43	4.13	12.22	0.236 (not significant)	[−18.9, 42.9]

## Data Availability

The raw data supporting the conclusions of this article will be made available by the authors on request.
